# Micro-bubble emission boiling with the cavitation bubble blow pit

**DOI:** 10.1038/srep33454

**Published:** 2016-09-15

**Authors:** Shigeaki Inada, Kazuaki Shinagawa, Suhaimi Bin Illias, Hiroyuki Sumiya, Helmisyah A. Jalaludin

**Affiliations:** 1 Dept. of Mechanical Science and Technology, Gunma University, Kiryu, Japan; 2 Kobelco Eco-Solutions Co., Ltd., Akasi, Hyogo Japan; 3 School of Manufacturing, Universiti Malaysia Perlis, Perlis, Malaysia; 4 Parker Co., Ltd., Ota, Gunma, Japan; 5 Faculty of Mechanical Engineering, Universiti Teknologi Mara, Terengganu Darul Iman, Malaysia

## Abstract

The miniaturization boiling (micro-bubble emission boiling [MEB]) phenomenon, with a high heat removal capacity that contributes considerably to the cooling of the divertor of the nuclear fusion reactor, was discovered in the early 1980s. Extensive research on MEB has been performed since its discovery. However, the progress of the application has been delayed because the generation mechanism of MEB remains unclear. Reasons for this lack of clarity include the complexity of the phenomenon itself and the high-speed phase change phenomenon in which boiling and condensation are rapidly generated. In addition, a more advanced thermal technique is required to realize the MEB phenomenon at the laboratory scale. To the authors’ knowledge, few studies have discussed the rush mechanism of subcooled liquid to the heating surface, which is critical to elucidating the mechanism behind MEB. This study used photographic images to verify that the cavitation phenomenon spreads to the inside of the superheated liquid on the heating surface and thus clarify the mechanism of MEB.

The International Thermonuclear Experimental Reactor (ITER)^1^ was constructed as an international collaboration with the aim of securing a sustainable energy source by the mid-twenty-first century. The design of the prototype nuclear fusion reactor “demonstration power plant (DEMO)” was also proposed to build upon the ITER. The technical problems^2^ associated with DEMO are being actively studied, particularly with regard to establishing a safer design for the divertor, which performs the role of controlling fast neutron irradiation and high heat flux.

The design of ITER incorporates a divertor structure consisting of a closed system using a water cooling tube, and various researchers are actively developing such designs. The following have been adopted as the cooling tube: a swirl tube^3^ that inserts the twisted tape into the cooling tube and a screw tube^4^ that carves the screw fin in the cooling tube. However, it is difficult to reduce the pump power necessary for the flow of cooling water when using such cooling pipes. Here, we propose a water cooling method in an open system with a superior heat removal ability compared to that of the cooling pipe. At present, the phenomenon with the highest heat removal ability is miniaturization boiling (micro-bubble emission boiling [MEB]).

MEB can be expected to be used not only for cooling of the divertor but also in application of the micro-cooling device. However, the mechanism of MEB generation and the process have not yet been elucidated because MEB generation in the highly subcooled liquid is a complex phenomenon that shows unique boiling behaviour in the unstable transition boiling region; in addition, a more advanced thermal technique is required to realize the MEB phenomenon at the laboratory scale.

Since the MEB phenomenon was first discovered^5^,^6^,^7^ in the early 1980s, research on the occurrence condition and heat transfer characteristics of MEB has been actively conducted^8^,^9^,^10^. Miniaturization boiling is realized in the boiling system, in which a subcooled liquid exists in the neighbourhood of the superheated liquid. The boiling is realized not only in the pool^6^,^7^,^11^,^12^,^13^ and forced convection boiling system^8^,^9^,^10^ but also in the droplet collision boiling system^14^,^15^. In addition, the existence of the MEB phenomenon has been confirmed not only on the copper block as a heating system but also on a thin wire^16^. However, there are still several researchers who insist that MEB is merely a unique boiling phenomenon restricted to special experimental equipment.

To the authors’ knowledge, the rush mechanism of subcooled liquid to the heating surface, which is critical to elucidating the mechanism of MEB, has only been discussed by Kubo, R. & Kumagai, S.^17^ and Ando, J. *et al*.^18^.

Kubo, R. & Kumagai, S.^17^ observed the MEB phenomenon by installing the upward copper block as a heating surface at the bottom of a container filled with the subcooled bulk liquid.

For this equipment, the suction nozzle was installed to promote upward bulk liquid convection induced by a vapour bubble ejected from the heating surface.

This research reported the existence of two heating surface temperature ranges. One is the temperature range that exhibits stormy MEB, and the other is the temperature range that exhibits calm MEB. According to their observations in the stormy MEB region, the constriction phenomenon of the tip of the bubble was photographed as the phenomenon that resembled the decay of the cavitation bubble in the vicinity of the heating surface. A grasp of this constriction phenomenon offers important evidence of the generation of the micro-jet.

Ando, J. *et al*.^18^ performed boiling experiments on a horizontal circular heating surface in a subcooled pool with a focus on the transition process of MEB. As a result of tracking the micro-bubble that arose in MEB, they observed a rush of subcooled liquid to the heating surface during the decay of the coalescence vapour bubble. Moreover, they found that the convection disturbance pattern under the MEB condition in the vicinity of the heating surface differs considerably from that of the typical nucleate boiling condition.

These two studies illustrate that it is difficult to explain the mechanism of MEB using only the three well-known factors of latent heat transportation, disturbance and liquid–vapour exchange in typical nucleate boiling. Another factor is likely involved in the MEB phenomenon.

Both of the aforementioned studies have presented useful demonstration data that prove the rush mechanism of subcooled liquid to the heating surface. However, neither have explained the liquid–solid contact behaviour and liquid–vapour exchange behaviour in the superheated liquid on the heating surface after the liquid rush.

The mechanism of the rush of subcooled liquid to the heating surface and the heat removal mechanism that the liquid rush induces must be clarified based on the visualization in the superheated liquid. In addition, it is necessary to verify that these two actions (liquid rush and heat removal) can be stably repeated.

## Experimental Apparatus and Procedure

**Figure 1:**
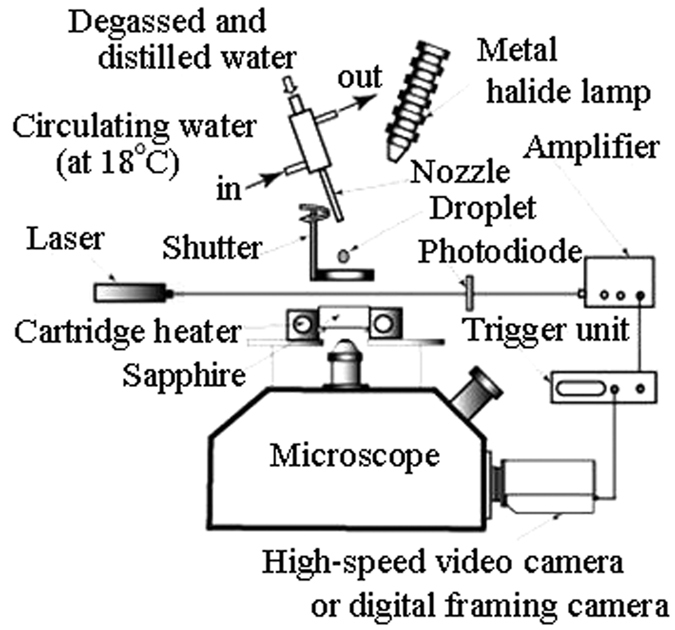
Experimental apparatus.

**Figure 2:**
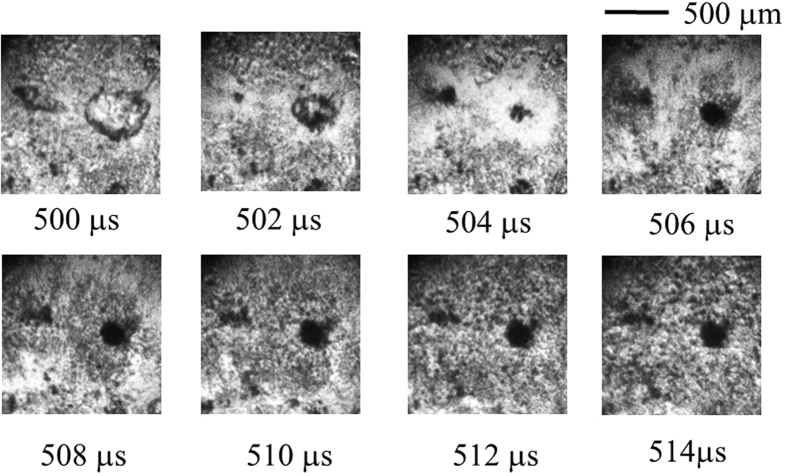
Boiling behaviour on the sapphire heating surface for *T*_wi_ = 320 °C visualized using a digital framing camera (500,000 fps). (See Supplementary Information Video, Supple-Video MEB(wmv)).

**Figure 3:**
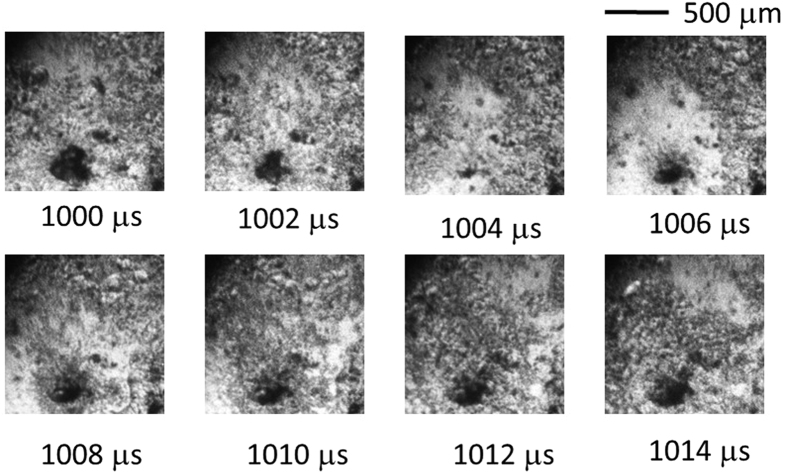
Boiling behaviour on the sapphire heating surface for *T*_wi_ = 320 °C visualized using a digital framing camera (500,000 fps). (See Supplementary Information Video, Supple-Video MEB(wmv)).

**Figure 4:**
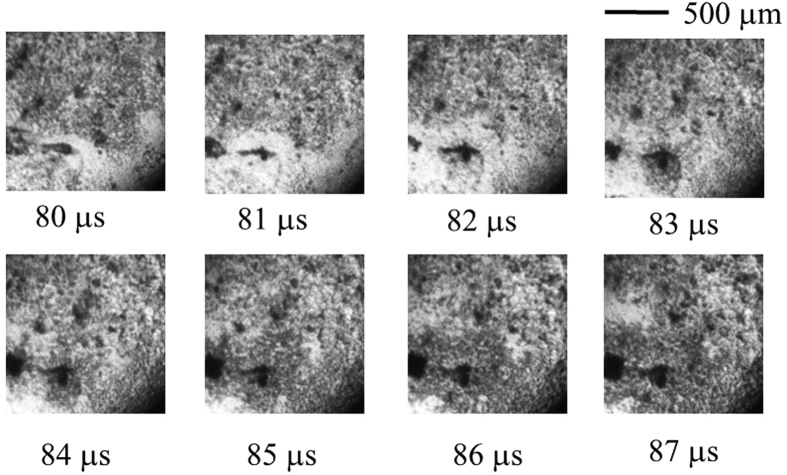
Boiling behaviour on the sapphire heating surface for *T*_wi_ = 320 °C visualized using a digital framing camera (1,000,000 fps). (See Supplementary Information Video, Supple-Video MEB(wmv)).

Figure 1 presents a schematic of the experimental apparatus, which was also used in a previous report^19^. An artificial and white transparent sapphire was used as the heating surface. The heating surface was a disk of 30 mm in diameter and 8 mm in height; (material, Al_2_O_3_; thermal conductivity, 46 W/m K), both sides of which were finished as specula with an accuracy equal to that of the lens, and it was maintained at a level position. The sapphire disk was inserted into the central part of the heated cylindrical copper block in which cartridge-type heaters are built such that the block could be uniformly heated from its periphery through heat conduction. Degassed and distilled water was used as a droplet. The water droplet diameter was maintained at 4.0 mm, and the falling height was kept at 65 mm. This height is in a range in which the droplet does not break itself apart via collision energy. Although the droplet collision Weber number defined using the diameter and falling height of the droplet is described below, the Weber number (We) is 70 in the photographs from the experiment shown in Figs 2, 3, 4.

The temperature of the droplet was kept at 16 °C by circulating tap water along the circumference of the nozzle. The temperature of the heating surface was measured by attaching the thermocouple with ceramic adhesive at two points on the surface. The behaviour of the microbubble was observed through a microscope at 4× and 10× magnification from the back surface of the sapphire disk. A digital framing camera that could record eight continuous frames was used to photograph the micro-bubble, and the frame rate was 500,000–1,000,000 fps. The output signal of the photodiode was used as the trigger when the droplet obstructed the laser beam in situations in which the laser beam had irradiated on the photodiode beforehand.

## Results and Discussion

The following phenomena can be confirmed based on detailed observations of the photographs shown in Figs 2, 3, 4. Figure 2 shows the image photographed by the digital framing camera in the case of *T*_wi_ = 320 °C. This initial heating surface temperature corresponds to the temperature range in which the most remarkable MEB phenomenon is observed. The time elapsed from when the droplet collided with the heating surface is shown under each photograph. The visual field photographed through the microscope is a narrow region including the droplet collision point. At the instant of droplet collision, the entrained air is captured by the confinement between the droplet and heating surface. The entrained air is absorbed in many vapour bubbles that are intensely created afterward, and then, it is immediately extinguished. In the elapsed time shown in Fig. 2, a scattering of a large number of minute vapour bubbles formed in the superheated liquid is observed, unlike in the smooth interface occupied solely by the superheated liquid. One large vapour bubble of approximately 0.5 mm in diameter, which grows by the coalescence of minute vapour bubbles, is observed to the right of the centre of Fig. 2 (at 500 μs). In the second frame (502 μs), the coalescing bubble is instantly contracted by the condensation action of the subcooled liquid that exists in the back side, which is perpendicular to the plane of view of the photograph.

After the decay and disappearance of the bubble, the subcooled liquid immediately rushes to the heating surface. The liquid that rushed from the central part where the bubble disappeared has left traces of the radial blowout (at 504 μs). The trace was brightly photographed because a bubble object that obstructed the lighting instantly disappeared. The disappearance of the bubble in such a situation appears to accompany the luminous phenomenon. However, the intensity of the light emission is extremely weak compared with the photographing light intensity.

After the rush of the subcooled liquid, many micro-bubbles spout from the rush site in succession. Here, the ‘rush of the subcooled liquid’ appears to be a phenomenon that is compatible with the ‘micro-jet’ or ‘liquid jet’ defined in the cavitation phenomenon. The many scattered microbubbles receive heat from the superheated liquid, and they individually increase the volume (from 506 μs onward).

Cavitation is a physical phenomenon in which expansion and contraction of a bubble are instantly generated by the pressure difference in the flow of the liquid. A collapse process of the cavitation bubble under the condition in which the bubble attached to a fixed surface traces the following progress. The apical surface of the bubble not attached to the fixed surface caves inward. The apical surface collides with the fixed surface at the speed of the jet, and the bubble collapses. The jet flow is called a micro-jet. When the micro-jet reaches the fixed surface and the bubble collapses, an impulsive force is added to the fixed surface.

The spouting position of the micro-bubble is shown in Fig. 2 as a dark, round pit. However, this form and size are dependent on those of the collapsing coalescing bubble. In Figs 3 and 4, the coalescing bubble is neither circular nor large. The phenomenon observed in the third frame (at 504 μs) of Fig. 2 corresponds to the third or fourth frames of Fig. 3 (at 1,004 μs or 1,006 μs) as a similarity phenomenon. The fourth frame (at 506 μs) of Fig. 2 corresponds to the fifth frame (at 1,008 μs) of Fig. 3. The following frames are similarly correspondent.

It is important to consider why the blow pit is observed in the dark state in these photographs. According to Lindau and Lauterborn^20^, the photograph illustrates the following phenomenon. At the start of cavitation, which is accompanied by a shock wave with bubble collapse, the collapsed bubble changes suddenly into small bubbles of a cloud state. In our experiment, the appearance of the blow pit as a dark hole appears to originate from the generation of small bubbles in the cloud condition, which is similar to the phenomenon reported by the aforementioned authors^20^. Because the bubbly flow under the cloud state rushes to the blow pit simultaneously, the lighting at that site is shielded. As a result, the blow pit is observed as a dark hole. An individual bubble in the cloud state expands under decompression with the flow, and it flows in the superheated liquid as the micro-bubble grows.

In our experiment, another occurrence that proves the generation of cavitation and the accompanying shock wave is the visualization of the shock waves emitted. Ohl *et al*.^21^ presented an image of the different shock waves emitted at the bubble collapse.

Figure 4 includes a trace that appears to be a compression shock front, similar to the shock front photographed by Ohl *et al*.^21^. A dark pit that is slightly separated from the centre of each photograph in Fig. 4 can be observed in the lower left. The form is similar to an airplane shape with spread wings. A shock wave emitted from the pit spreads in an oval conformation, and the blowing of the micro-bubble is confirmed.

The noise is generated by the volume variation that originates from the decay and contraction of the cavitation bubble when the cavitation is generated. We then calculate the collapse time of a bubble using Rayleigh’s equation and estimate the reciprocal of the collapse time as a frequency of the noise. The noise frequency is approximately 10 kHz when the bubble radius at collapse is 1 mm and approximately 6.7 kHz when the radius is 1.5 mm. Inada and Yang^15^ measured the boiling sound range (8–9 kHz), which was approximately the aforementioned noise frequency, when a droplet impinged on the heating surface, where the initial wall temperature was kept within the range of 260–340 °C. The boiling sound was measured using a quartz piezoelectric device installed on the heating surface as an elastic longitudinal wave that propagates along the heating surface. In droplet boiling with such a high noise frequency, a violent dispersion of minute liquid clusters into the surrounding atmosphere was observed. This phenomenon is called the ‘miniaturization of a sessile drop’.

In a subsequent study^22^, Inada and Yang evaluated the heat transfer characteristic in two subcooled boiling systems: minute droplet dispersion boiling and minute vapour bubble dispersion boiling. The former is realized in a droplet boiling system under a non-steady state, and the latter is realized under a steady state in a pool boiling system in which the heating surface was submerged in the subcooled liquid. In the former experiment, for example, when the initial surface temperature is first set in the range of 260–340 °C, the transient temperature of the heating surface ranges within 180–200 °C during the period in which a violent dispersion of minute liquid clusters is observed. Although this temperature range is affected by the droplet subcooling and Weber number, the changes are relatively minor. However, the heat flux removed by the droplet from the heating surface is affected by the droplet subcooling and Weber number. The droplet subcooling is the temperature difference between the saturation temperature and droplet temperature of the water under atmospheric pressure, and it is denoted as Δ*T*_sub_. In addition, the subcooling in pool boiling is the temperature difference between the saturation temperature and pool water bulk temperature under atmospheric pressure, denoted as Δ*T*_sub_.

The Weber number is a dimensionless number defined as the ratio of inertial force to the surface tension—that is, *We* = ρ*ν*^2^*d*/*σ*, where ρ is the density of the droplet, *ν* is the droplet impinging velocity, *d* is the droplet diameter and *σ* is the surface tension of the droplet.

To compare the heat flux of the two boiling systems mentioned in the preceding text, the transient heating surface temperature *T*_*w*_ and heat flux q_*w*_ were estimated based on the real-time temperature information measured by four thermocouples installed on the central axis of a cylindrical heat conduction rod of the conical copper block^23^,^24^. The heating surface temperature was calculated by the two-dimensional finite differential method based on the measured temperature information. The heat flux was estimated by solving the inverse problem of two-dimensional unsteady heat conduction. Here, the heating surface temperature of the wetting interface with the droplet was assumed to be uniform.

**Figure 5:**
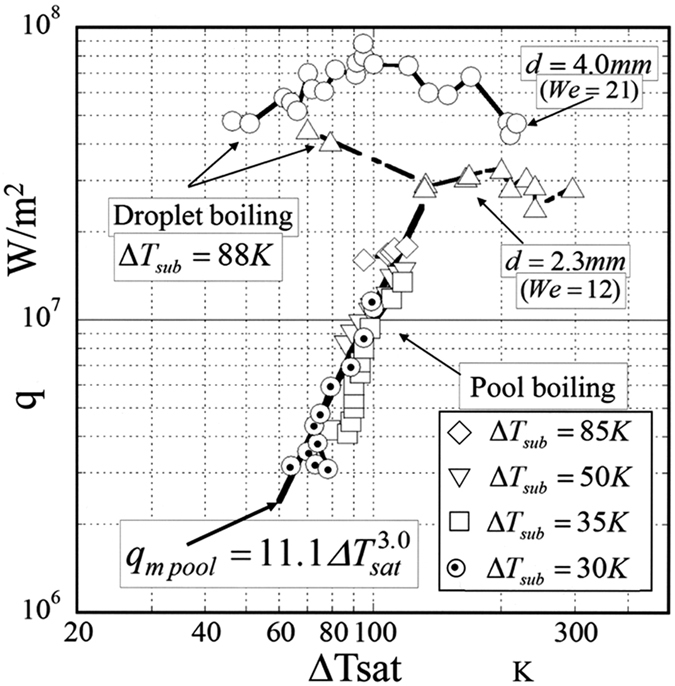
Boiling curves for MEB in two boiling systems using the copper block heating surface.

The heating surface temperature and heat flux plotted in Fig. 5 were evaluated as the mean time value defined by the following equations^23^,^24^:


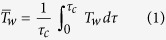



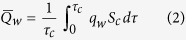



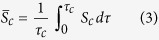



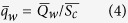


where *S*_*c*_ is the spread area of a droplet bottom, which makes contact with the heating surface and is estimated from the transient drop bottom radius profiles. The upper bound of the integration is referred to as the effective heat transfer period *τ*_*c*_ and is estimated to be approximately 50–60% of the residence time of a droplet on the heating surface, with the residence time approximately equal to the first free-vibration period of a droplet.

Figure 5 shows the characteristic curves of the miniaturization boiling for two boiling systems. One represents four different subcoolings: Δ*T*_sub_ = 30, 35, 50 and 85 K in the pool boiling. The correlation curve of the MEB in pool boiling previously proposed by Inada *et al*.^11^,^12^ is shown as a bold solid line in Fig. 5. Another curve represents two different droplet diameters, 2.3 and 4 mm, with the same subcooling Δ*T*_sub_ = 88 K. The temperature difference between the time mean temperature by Equation (1) and the saturation temperature of water under atmospheric pressure—that is, the wall superheat Δ*T*_sat_—has been adopted on the horizontal axis of Fig. 5, whereas the heat flux defined by Equation (4) has been adopted on the vertical axis.

Compared with the measured heat flux shown in Fig. 5 for the two boiling systems, the heat flux of 81 MW/m^2^ has been obtained at a wall superheat Δ*T*_sat_ = 100 K in the droplet boiling system, where *d* = 4.0 mm, the droplet Weber number *W*_*e*_ = 21 and the droplet subcooling Δ*T*_sub_ = 88 K. In contrast, a heat flux of 11 MW/m^2^ has been obtained at Δ*T*_sat_ = 100 K in the pool boiling system. The heat flux in the droplet boiling system is approximately 7.4 times larger than that in the pool boiling system.

According to the correlation equation in the pool boiling system shown in Fig. 5, the heat flux increases with increasing Δ*T*_sat_, but there is an upper limit called the second burnout heat flux^12^. The upper limit of the heat flux is approximately 18 MW/m^2^ for Δ*T*_sub_ = 91.8 K and Δ*T*_sat_ = 117.6 K. Thus, the adoption of the droplet boiling system with the MEB phenomenon is indispensable to ensure that the heat flux exceeds 18 MW/m^2^. In the droplet boiling system, it can be sufficiently expected that a heat flux that exceeds the value shown in Fig. 5 will be ensured by further increasing the Weber number.

However, the heat transfer enhancement is not always good when the Weber number is large. When the droplet collided with the heating surface, an impinging speed at which the droplet itself does not break apart and does not block the dispersion of the minute liquid clusters generated at the bubble collapse is required.

The MEB phenomenon with the cavitation must be steadily realized in the droplet boiling system to ensure a heat flux of over 30 MW/m^2^, which contributes to the cooling of the divertor.

One method is to continuously bump the swarms of droplets on the heating surface. However, the bumping flow rate of the droplet swarm must be controlled to prevent the submergence of the heating surface with increases in the water bulk.

A vapour bubble that takes the vaporization heat from the heating surface must leave the field rapidly to steadily ensure the high heat transfer characteristic.

When comparing with the measured heat fluxes shown in Fig. 5 for the two boiling systems, minute droplet dispersion boiling and minute vapour bubble dispersion boiling, the high heat transfer characteristic was obtained in the former system. This result was obtained because the environmental condition for boiling, in which the heat transportation vapour bubble can continuously and rapidly scatter from the heating surface to the atmosphere, is superior in the minute droplet dispersion boiling system compared to the minute vapour bubble dispersion boiling system.

## Conclusions

The phenomena described in the following steps, which elucidate the mechanism of MEB, were clarified based on the results obtained using high-speed photographic images of the boiling phenomenon in a droplet boiling system:

1. An individual micro-bubble is coupled one after another in the superheated liquid and grows to one large coalescing bubble; then, the coalescing bubble is projected into the subcooled liquid.
2. The coalescing bubble that is projected into the subcooled liquid is rapidly contracted by condensation of the subcooled liquid.
3. At this time, the coalescing bubble instantaneously collapses with the micro-jet. This micro-jet brings about a rush of subcooled liquid to the heating surface.
4. A large number of micro-bubbles that arose due to the cavitation are blown out in the superheated liquid through the blow pit.
5. In the superheated liquid, thermal energy is supplied to the micro-bubble, and the volume is individually increased.

Each of the aforementioned phenomena is stably repeated in this order.

In summary, the exchange of vapour and liquid on the heating surface is activated by the collapse of the vapour bubble with the cavitation that arises at the interface between the superheated liquid and subcooled liquid. As a result, a high heat flux is obtained. The boiling system must be constructed without blocking the violent dispersion of minute liquid clusters generated at the bubble collapse.

## An Analogy and Projection

For the realization of a nuclear fusion reactor in an early stage, we anticipate that the droplet collision cooling method with the MEB phenomenon would be applied to the divertor cooling system.

To take an example from astronomy, the mechanism of formation of the blow pit that is created by the cavitation bubble observed in this study may be seen as analogous to the mechanism of the formation of a crater on the lunar surface. Researchers knowledgeable in astronomy may evaluate the appropriateness of the following inferences. We consider the lunar surface during the magma ocean stage when the magmatic viscosity was extremely high. The magma, which included various gases, gushed out in the bubble state from the magma ocean surface. The spouting bubble was cooled instantaneously such that cavitation was generated by rapid contraction of the bubble. The apical surface of the bubble collided with the magma ocean surface with impulsive force as the micro-jet. The crater was formed as a result.

## Additional Information

**How to cite this article**: Inada, S. *et al*. Micro-bubble emission boiling with the cavitation bubble blow pit. *Sci. Rep.* **6**, 33454; doi: 10.1038/srep33454 (2016).

## Supplementary Material

Supplementary Information

Supplementary Video
