# Supplementary material for: Micro-bubble emission boiling with the cavitation bubble blow pit
Source: Sci Rep. 2016 Sep 15;6:33454. doi: 10.1038/srep33454 (PMC5024302; doi:10.1038/srep33454)
Supplement: Supplementary Information [file srep33454-s1.pdf]

Supplementary Information File

1. Title of the manuscript:

Micro-bubble emission boiling with the cavitation bubble blow pit

2. Author list:

Shigeaki Inada*[1], Kazuaki Shinagawa[2], Suhaimi Bin Illias[3], Hiroyuki Sumiya[4], & Helmisyah A. Jalaludin[5]

Primary affiliation: 1-5,

Dept. of Mechanical Science and Technology, Gunma University, Kiryu 376-8515, Japan.

Corresponding author: *[1]Shigeaki Inada (E-mail: inada-plasma@outlook.com)

Author information

Shigeaki Inada: Corresponding author.

Emeritus Professor of Gunma University.

(Home address): 376-0011, Aioi-cho, 1-406-19, Kiryu City, Gunma Pref., Japan.

E-mail: inada-plasma@outlook.com

Telephone: 080-1180-5226, or 0277-55-5017

Fax: 0277-55-5017

Kazuaki Shinagawa

Kobelco Eco-Solutions Co., Ltd. 674-0092, Higashifutami, 482-3, Futami-cho, Akasi City, Hyogo Pref., Japan.

E-mail: k.shinagawa@kobelco-eco.co.jp; skashwing3@yahoo.co.jp

Suhaimi Bin Illias

Senior Lecturer of Universiti Malaysia Perlis.

School of Manufacturing Engineering, Universiti Malaysia Perlis, Pauh Putra Campus, 02600 Arau, Perlis, Malaysia.

E-mail: suhaimi@unimap.edu.my

Hiroyuki Sumiya

Parker Co., Ltd.

373-0806, Ryumai-cho, 5320, Oto City, Gunma Pref., Japan.

E-mail: sumiya@parkercorp.co.jp

Helmisyah Ahmad Jalaludin
Lecturer of University Teknologi Mara.
Faculty of Mechanical Engineering, Universiti Teknologi Mara (Terengganu), Bukit Besi Campus, 23200 Bukit Besi, Terengganu Darul Iman, Malaysia.
E-mail: helmisyah@tganu.uitm.edu.my

3. **Supplementary Video**

Video file name: "Supple-Video MEB (mov)"

Video size: 1.9MB

Video legend: Droplet boiling behavior photographed by a high-speed video camera from the back surface of the heated sapphire disk (T_{wi} = 320 °C)
